# Association between SARS-CoV-2 infection and Kawasaki-like multisystem inflammatory syndrome: a retrospective matched case–control study, Paris, France, April to May 2020

**DOI:** 10.2807/1560-7917.ES.2020.25.48.2001813

**Published:** 2020-12-03

**Authors:** Julie Toubiana, Corinne Levy, Slimane Allali, Camille Jung, Marianne Leruez-Ville, Emmanuelle Varon, Fanny Bajolle, Naim Ouldali, Judith Chareyre, Stephane Béchet, Annie Elbez, Jean-Laurent Casanova, Martin Chalumeau, Robert Cohen, Jérémie F Cohen

**Affiliations:** 1 Department of General Pediatrics and Pediatric Infectious Diseases, Necker-Enfants Malades University Hospital, Assistance Publique – Hôpitaux de Paris (AP-HP), Université de Paris, Paris, France; 2 Institut Pasteur, Biodiversity and Epidemiology of Bacterial Pathogens, Paris, France; 3 ACTIV, Association Clinique et Thérapeutique Infantile du Val-de-Marne, Créteil, France; 4 Université Paris Est, IMRB-GRC GEMINI, Créteil, France; 5 Clinical Research Center (CRC), Centre Hospitalier Intercommunal de Créteil, Créteil, France; 6 GPIP, Groupe de Pathologie Infectieuse Pédiatrique, Paris, France; 7 AFPA, Association Française de Pédiatrie Ambulatoire, Saint-Germain-en-Laye, France; 8 Virology laboratory, Necker-Enfants Malades University Hospital, AP-HP, EA 7328, Université de Paris, Paris, France; 9 Laboratoire de Microbiologie, Centre Hospitalier Intercommunal de Créteil, Créteil, France; 10 M3C-Necker-Enfants Malades University Hospital, AP-HP, Université de Paris, Paris, France; 11 Unité d’épidémiologie clinique, AP-HP, Hôpital Robert Debré, ECEVE INSERM UMR 1123, Paris, France; 12 Pediatric Intensive Care Unit, Necker-Enfants Malades University Hospital, AP-HP, Université de Paris, Paris, France; 13 Laboratory of Human Genetics of Infectious Diseases, Necker Branch, Imagine Institute, Pediatric Hematology and Immunology Unit, Necker-Enfants Malades University Hospital, Université de Paris, Paris, France; 14 St. Giles Laboratory of Human Genetics of Infectious Diseases, Rockefeller Branch, The Rockefeller University, Howard Hughes Medical Institute, New York, United States; 15 Université de Paris, Centre of Research in Epidemiology and Statistics - CRESS, INSERM, F-75004 Paris, France; 16 Unité Court Séjour, Petits nourrissons, Service de Néonatalogie, Centre Hospitalier Intercommunal de Créteil, Créteil, France

**Keywords:** Kawasaki-like disease, PIMS-TS, MIS-C, SARS-CoV-2, COVID-19

## Abstract

We assessed the association between severe acute respiratory syndrome coronavirus 2 (SARS-CoV-2) infection and Kawasaki disease (KD)-like multisystem inflammatory syndrome in a retrospective case–control study in France. RT-PCR and serological tests revealed SARS-CoV-2 infection in 17/23 cases vs 11/102 controls (matched odds ratio: 26.4; 95% confidence interval: 6.0–116.9), indicating strong association between SARS-CoV-2 infection and KD-like illness. Clinicians should keep a high level of suspicion for KD-like illness during the COVID-19 pandemic.

Multisystem inflammatory syndrome in children and adolescents (MIS-C) emerged during the coronavirus disease (COVID-19) pandemic, leading to a first alert by the United Kingdom National Health Service on 25 April 2020 [1]. Since then, several case studies in regions with high rates of severe acute respiratory syndrome coronavirus 2 (SARS-CoV-2) community transmission have reported MIS-C cases [2], with a substantial proportion of patients meeting the American Heart Association criteria for Kawasaki disease (KD) [3-5]. We investigated this potential association in the Paris metropolitan area (Île-de-France), France, using a matched case–control design.

## Definition of cases and controls and matching

Our retrospective case–control study using patient data from two previous studies [3,6] covered the period from 14 April to 26 May 2020.

Cases with KD-like illness were children and adolescents (≤ 18 years) fulfilling the American Heart Association criteria for complete (fever > 4 days and ≥ 4 principal criteria) or incomplete (fever > 4 days and 2 or 3 principal criteria, and without characteristics suggestive of another diagnosis) KD [7], admitted to the general paediatric department of Necker-Enfants malades referral hospital [3]. Each case was matched randomly to a maximum of five controls sampled without replacement from a prospective multicentre cross-sectional study conducted in 27 primary care paediatric private practices in the Paris area during the same period [6]. In brief, controls were children (≤ 15 years), symptomatic or not, visiting one of the paediatricians from the Association Clinique et Thérapeutique Infantile du Val de Marne (ACTIV) network. Cases and controls were matched by ZIP code (exact matching of the first two numbers) and age (± 2 years).

## Laboratory investigations

For each child/adolescent, nasopharyngeal swabs were obtained to test for SARS-CoV-2 using reverse transcription-PCR (RT-PCR). Cases were tested using the SARS-CoV-2 R-GENE test (bioMerieux, Marcy-l’Etoile, France); controls were tested with the Allplex 2019-nCoV assay (Seegene, Seoul, South Korea). Positivity for RT-PCR was considered consistent with recent or ongoing SARS-CoV-2 infection.

All children/adolescents were also tested for antibodies against SARS-CoV-2. Cases were tested using the Architect SARS-CoV-2 assay (Abbott Core Laboratory, Illinois, United States (US)), a chemiluminescent immunoassay for the quantitative detection of IgG antibodies in serum. Controls were tested using the COVID-19 BSS test (Biosynex, Strasbourg, France) [8], a rapid chromatographic immunoassay for the qualitative detection of IgG antibodies on fingerstick whole-blood specimens. These tests were both evaluated by the French National Reference Centre for respiratory viruses at the Institut Pasteur, Paris and were among those approved by the French national health authority (Haute Autorité de Santé), Saint-Denis. Positive serology was defined as the detection of IgG against SARS-CoV-2 and was deemed consistent with recent infection with SARS-CoV-2 [9].

## Analyses

We described baseline characteristics of cases and controls and performed conditional logistic regressions to calculate matched odds ratios (ORs) to assess the association between SARS-CoV-2 infection and KD-like illness. We tested the association in the following subgroups: (i) overall positive (positive RT-PCR and/or serology), (ii) RT-PCR positive (independently of the serology result) and (iii) serology positive (independently of the RT-PCR result). We conducted two sensitivity analyses: (i) matching by ZIP code, age and sex, and (ii) matching by ZIP code, age and exact week of inclusion. Statistical analyses were conducted using Stata/SE 15 (StataCorp, College Station, US).

## Ethical statement

For cases, the study protocol was approved by the Necker Hospital Institutional Review Board (No. 20200618174239) and by an ethical committee (CPP Ouest IV, No. DC-2017–2987). For controls, the study protocol was approved by an ethical committee (CPP IDF IX, Number 08–022) and was registered at ClinicalTrials.gov (NCT04318431). All parents provided written informed consent. This study was conducted in accordance with the Helsinki Declaration.

## Association between SARS-CoV-2 infection and Kawasaki-like multisystem inflammatory syndrome

Among 30 eligible cases (i.e. all cases with KD-like illness hospitalised in our general paediatric department) and 605 controls already described elsewhere [3,6], the matching process could not identify controls for seven cases; these were therefore excluded from the case–control study. For the remaining 23 cases, the matching process allowed for pairing one control for one case, two controls for two cases, three controls for one case, four controls for one case and five controls for 18 cases, respectively. Thus, analyses relied on data for 23 cases with KD-like illness (mean age 6.8; range: 0.3–16.6 years) and 102 controls (mean age 5.8; range: 0.05–16.0 years), slightly less than half of whom were females (Figure and Table **)**.

**Figure:**
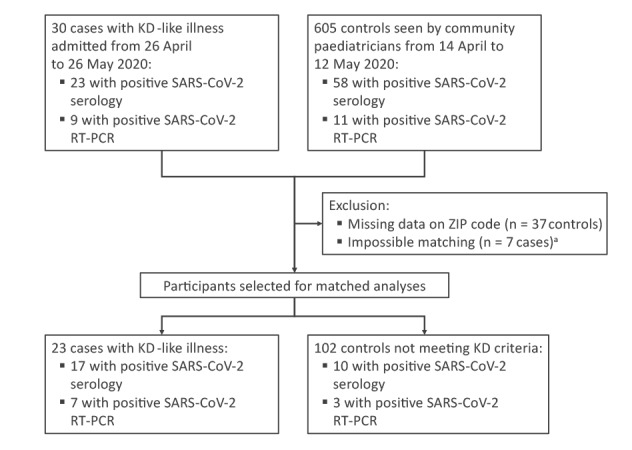
Flowchart of study participant selection

KD: Kawasaki disease; RT-PCR: reverse transcription-PCR; SARS-CoV-2: severe acute respiratory syndrome coronavirus 2.

^a^ Each case was matched to a maximum of five controls, with matching on ZIP code and age. Among 30 eligible cases with KD-like illness, seven could not be matched with any control and were excluded.

**Table:** Characteristics of cases with Kawasaki disease-like illness and controls (n = 125)

Characteristics	Cases (n = 23)		Controls (n = 102)		Matched OR (95% CI)^a^
	Number	%	Number	%	
**Sex, females**	11	48	48	47	NA
**Age, mean in years (SD)**	6.8 (4.8)	NA	5.8 (4.1)	NA	Matching criterion
**Name of Department within Paris metropolitan area (first two digits of ZIP code)**					Matching criterion
Paris (75)	4	17	16	16	NA
Seine-et-Marne (77)	2	9	10	10	
Yvelines (78)	1	4	2	2	
Essonne (91)	2	9	7	7	
Hauts-de-Seine (92)	4	17	20	20	
Seine-Saint-Denis (93)	6	26	27	26	
Val-de-Marne (94)	4	17	20	20	
**Overall evidence of SARS-CoV-2 infection i.e. positive RT-PCR and/or serology**					
Positive	17	74	11	11	26.4 (6.0–116.9)
Negative	6	26	91	89	Reference
**SARS-CoV-2 RT-PCR testing independently of the serology result**					
Positive	7	30	3	3	13.9 (2.8–68.6)
Negative	16	70	99	97	Reference
**SARS-CoV-2 serology testing independently of the RT-PCR result**					
Positive	17	74	10	10	27.7 (6.3–122.7)
Negative	6	26	92	90	Reference

CI: confidence interval; NA: not applicable; OR: odds ratio; RT-PCR: reverse transcription-PCR; SD: standard deviation.

^a^ Conditional logistic regression with patients matched on ZIP code and age.

Overall, 17 of 23 (74%) cases and 11 of 102 (11%) controls tested positive for SARS-CoV-2 by RT-PCR and/or serology (matched OR: 26.4; 95% confidence interval (CI): 6.0–116.9). The association remained significant when limiting the assessment to RT-PCR results and serological results separately (matched OR: 13.9; 95% CI: 2.8–68.6 and 27.7; 95% CI: 6.3–122.7, respectively; **Table** **)**. The association was robust in sensitivity analyses with matching on ZIP code, age and sex (21 cases vs 88 controls; matched OR: 21.6; 95% CI: 4.8–97.1), as well as on ZIP code, age and week of inclusion (12 cases vs 38 controls; matched OR: 24.3; 95% CI: 3.0–198.5).

## Discussion and conclusions

During the COVID-19 pandemic, an outbreak of MIS-C with features of myocarditis, toxic shock syndrome and KD was reported by several teams [2], including ours [3]. An association between SARS-CoV-2 infection and this KD-like illness has been hypothesised, based on the 2 to 4 weeks delay between the peak of SARS-CoV-2 infections and that of KD-like illness cases in the populations studied [2,3]. However, because these studies lacked control groups without KD-like illness, they could not formally test this hypothesis. To date, a causal link between viral infections and the occurrence of KD remains probable but unproven. Several viral pathogens, such as Epstein-Barr virus [10], cytomegalovirus [11], parvovirus B19 [12] and bocavirus [13], have been suggested as possibly involved in the pathogenesis of KD, but the association with coronaviruses remains debated because of conflicting results [14]. Further to the evidence from observational case series, this study provides strong evidence of an association between infection with SARS-CoV-2 and the occurrence of KD-like illness observed during the COVID-19 pandemic, with 25-fold higher odds of exposure to SARS-CoV-2 in patients with KD-like illness.

This study has several strengths. First, we focused on patients fulfilling criteria for KD [7], as MIS-C definitions can potentially bring clinical heterogeneity [15,16]. Second, cases and controls were included over the same period and geographical area, which reduces confounding due to spatiotemporal variations of exposure to SARS-CoV-2. Lastly, we conducted our research in the Paris metropolitan area, a region heavily affected by COVID-19, which enabled us to include enough cases.

This study also has limitations. First, despite matching by ZIP code, age, sex and week of inclusion, we cannot exclude residual confounding due to socioeconomic and ethnic factors. Indeed, some populations might have higher exposure to SARS-CoV-2 infection or particular genetic predispositions [3]. Unfortunately, these data were not available in controls. Second, Necker-Enfants malades hospital hosts a referral centre for paediatric cardiovascular disease and a large intensive care facility; thus, we may have included the most severe cases of KD-like illness. This potential selection bias may have led to an overestimation of the odds ratio. Third, serology and RT-PCR assays used in cases and controls were different, and we could not exclude measurement bias. Fourth, this study was conducted during the national lockdown and relied on a small sample of children/adolescents recruited in a limited geographical area. Our findings should be validated in larger international studies and in periods of more active community transmission of the virus.

In conclusion, we provide evidence of a strong association between SARS-CoV-2 infection and KD-like illness during the COVID-19 pandemic. Clinicians should be aware of this association and keep a high level of suspicion for KD-like illness, notably in children with clinical or laboratory evidence of recent infection with SARS-CoV-2.
